# Supplementary material for: Association between metabolic risk factors and optic disc cupping identified by deep learning method
Source: PLoS One. 2020 Sep 17;15(9):e0239071. doi: 10.1371/journal.pone.0239071 (PMC7498045; doi:10.1371/journal.pone.0239071)
Supplement: Attachment — Submitted filename: response_to_reviewers.docx [file pone.0239071.s002.docx]

**Point-by-Point Replies to Reviewer’s Comments**

Date: July 6. 2020

Dear Editor,

We appreciate the reviewers for their efforts and the constructive comments. A point-by-point response to each of reviewers’ comments follows. The authors wish to thank the Editorial Board for their thoughtful consideration and recommendations, and hope that this revised manuscript now meets your requirements for publication.

<Reviewer #1>

There are only a few grammatical errors to correct.

In the abstract line 23 and 24, reword it to say 'with over 20 year-old patients THAT underwent health screening examinations'
Line 134: Should it read 'HDL was categorized as follows' with an 's' at the end of 'follow' ?
Line 136: divided into 2 'groups' instead of 'group'
Line 203: Add 'a' to 'BMI had positively correlated with VCDR, and was also a risk factor for GON.'

🡺 As your comments, we corrected grammatical errors in manuscript.

<Reviewer #2>

The one major challenge with this paper was the obvious class imbalance. There was an almost 17:1 ratio of healthy subjects to GON subjects used in the statistical analysis. The authors should address this obvious class imbalance, and perhaps discuss the limitations of their models’ predictions given this fact. Other attempts to resolve this could include incorporating a focal loss cost function into their statistical methods.

🡺 We fully agreed with your comments. Unfortunately, the statistical consultant can’t suggest the definite solution about the obvious imbalance, so we described this imbalance between two groups in the limitation as following sentences;
 “Fifth, since the participants to undergo health screening examination enrolled in the health-care centre, the imbalance between normal subjects and study group was inevitably occurred in the present study.”

To allow readers to better understand what the authors meant by the YOLO deep learning model, a system diagram detailing the chosen deep learning model architecture is required. The diagram should include abbreviated number of layers, feature maps, input and output. The trained model with weights should be included as a supplemental file or a link that can be accessed (I know models tend to be massive in size).

🡺 As your comments, we attached the trained model as a supplemental file. After submitting this paper, the study about automatically measuring cup-to-disc ratio using deep-learning approach was published in ***Scientific Reports***. (Park K, Kim J, Lee J. Automatic optic nerve head localization and cup-to-disc ratio detection using state-of-the-art deep-learning architectures. Scientific Reports. 2020.10:5025. doi.org/10.1038/s41598-020-62022-x). We can’t suggest more detailed algorithm of measuring CDR because of commercial availability.

An additional system diagram detailing the flow of the entire process, from raw to final predictions would also be helpful for the reader to visualize the authors’ transition from raw clinical data to mathematical and clinical insights.

🡺 We fully agreed with your suggestion. Because the previous flow chart didn't include the detailed contents of metabolic risk factors, we added and revised the flow chart in Figure 1.

In regard to the statistical methods, there need to be clear mathematical equations, especially for the logistic/linear regressions. Describe reasons for using each statistical model. It was unclear if authors used a one tailed or two tailed t-test.
🡺 As your comments, we clearly explained the mathematical equations for logistic regression model in the table 4 and manuscripts as following sentences.

|  | Univariate | | Mutivariate^a^ | |
| --- | --- | --- | --- | --- |
|  | OR (95% CI) | P | OR (95%) | P |
| Age | 1.053 (1.046, 1.060) | <0.001 | 1.054 (1.046, 1.063) | <0.001 |
| Gender  (reference: Male) | 0.674 (0.584,0.779) | <0.001 | 0.708 (0.587, 0.854) | <0.001 |
| Body mass index (reference < 25 kg/m^2^) | 1.214 (1.055, 1.397) | 0.007 | 1.267 (1.065, 1.507) | 0.007 |
| Blood pressure (SBP / DBP) (reference < 130 / 80 mmHg) | 1.376 (1.174, 1.613) | <0.001 | 1.068 (0.867, 1.317) | 0.534 |
| Waist circumference (reference < 90 cm or men, <85 for women) | 1.099 (0.957, 1.263) | 0.082 | 1.025 (0.856, 1.227) | 0.790 |
| Triglyceride (reference < 150 mg/dL) | 0.933 (0.799, 1.089) | 0.378 | 0.845 (0.694, 1.030) | 0.086 |
| HDL cholesterol (reference ≥ 40mg/dL for men, ≥ 50 mg/dL for women) | 0.997 (0.991, 1.002) | 0.916 | 0.981 (0.787, 1.224) | 0.867 |
| HbA1c (reference < 6.5 %) | 2.274 (1.769, 2.922) | <0.001 | 1.606 (1.237, 2.086) | < 0.001 |
| Intraocular pressure | 1.016 (0.993, 1.039) | 0.099 | 1.016 (0.986, 1.048) | 0.299 |

^a^ By backward stepwise logistic regression model

“Univariate logistic analyses revealed that GON was significantly associated with older age, male gender, higher BMI, higher BP, and higher HbA1c (P < 0.001, < 0.001, 0.007, < 0.001, < 0.001, respectively). Those variables were evaluated in multivariable models with the covariates, using backward stepwise selection to eliminate those with P values > 0.05.”

For results, the authors should not only include correlation coefficients, but also include the specificity and sensitivity (in the form of an F1 score perhaps). A confusion matrix would help readers visualize how univariate or multivariate logistic models performed in predicting GON**.
🡺** We fully agreed with your comments. So, we added the specificity and sensitivity of the predicted probability as following sentence.
“ in logistic regression results, the predicted probability to discriminate between normal and GON showed 89.2 % specificity and 27.2 % sensitivity.”
