# Supplementary material for: Association between metabolic risk factors and optic disc cupping identified by deep learning method
Source: PLoS One. 2020 Sep 17;15(9):e0239071. doi: 10.1371/journal.pone.0239071 (PMC7498045; doi:10.1371/journal.pone.0239071)
Supplement: Attachment — Submitted filename: response_to_reviewers_2nd_revision.docx [file pone.0239071.s003.docx]

**Point-by-Point Replies to Reviewer’s Comments**

Date: August 21. 2020

Dear Editor,

We appreciate the reviewers for their efforts and the constructive comments. A point-by-point response to each of reviewers’ comments follows. The authors wish to thank the Editorial Board for their thoughtful consideration and recommendations, and hope that this revised manuscript now meets your requirements for publication.

<Reviewer #2>

1. Please also include the email of the IRB for data access (most researchers don't have access to international calls)

🡺 The study protocol was approved by the Pusan National University Yangsan Hospital Institutional Review Board and you can access for data through below email address [pnuyhirb@gmail.com](mailto:pnuyhirb@gmail.com).

2. Figure 2 has "vertical cup disk ratio" on the y axis. Is this correct, or is it the number of samples?

🡺 We make a mistake indexing on the y axis. As you would expect, index on the y axis was the number of samples. So, we changed the figure 2 as below.

3. Please explain the significance of a 89.2 % specificity and 27.2 % sensitivity. There is a stark contrast between the true positive and true negative rate; this needs to be discussed.

🡺 We fully agreed with your comments. As your comment in first revision, we previously analyzed the specificity and sensitivity for predicting GON by using multivariate logistic model, and described the 89.2% specificity and 27.2% sensitivity in the manuscript. Since cut-off value was set up as 0.5 automatically in SPSS program, we did not try calculating the various predicted probability as cut-off value was ranged from 0 to 1.0. According to the statistical consultant’s advices (Mi Sook Yoon PhD., msyun@pusan.ac.kr), we analyzed the all available parameters (accuracy, sensitivity, specificity, precision) showing the predicted probability in the range from 0.3 to 1.0 of cut-off value as below.

| cutoff | Corrected | | Incorrected | | **Accuracy** | **Sensitivity** | **Specificity** | **Precision** |
| --- | --- | --- | --- | --- | --- | --- | --- | --- |
|  | Normal | glaucoma | Normal | glaucoma |  |  |  |  |
| 0.3 | 8942 | 1609 | 2112 | 2045 | 71.7% | 43.2% | 81.4% | 84.8% |
| 0.4 | 9824 | 1034 | 1984 | 1866 | 73.8% | 34.3% | 84.0% | 90.5% |
| **0.5** | **11046** | **631** | **1690** | **1341** | **79.4%** | **27.2%** | **89.2%** | **94.6%** |
| 0.6 | 12155 | 104 | 1572 | 877 | 83.3% | 6.2% | 93.3% | 99.2% |
| 0.7 | 14708 | 0 | 877 | 0 | 94.4% | 0.0% | 100.0% | 100.0% |
| 0.8 | 14708 | 0 | 877 | 0 | 94.4% | 0.0% | 100.0% | 100.0% |
| 0.9 | 14708 | 0 | 877 | 0 | 94.4% | 0.0% | 100.0% | 100.0% |
| 1 | 14708 | 0 | 877 | 0 | 94.4% | 0.0% | 100.0% | 100.0% |

The low value of sensitivity in our study may be influenced by the parameters analyzed in logistic regression method, and could be improved as adding the useful diagnostic factors as the independent parameters in logistic regression analysis. In fact, the statistical results showed that the value of sensitivity was increased as the cut-off value was lower than 0.5, and then we should investigate the further evaluation including the ROC analysis.

In some studies of ophthalmogy *(Sharma, et al. Post penetrating keratoplasty glaucoma: Cumulative effect of quantifiable risk factors. DOI: 10.4103/0301-4738.129790 / Lu et al. Comparison of Ocular Biomechanical Machine Learning Classifiers for Glaucoma Diagnosis. DOI: 10.1109/BIBM.2018.8621238)*, the goodness-of-fit of logistic regression models uses the accuracy of the classification table. Moreover, as your comments, there was outstanding gap between the sensitivity and specificity rate. Then, we and statistical consultant determined that the accuracy rate could be the appropriate parameter as probability for predicting GON.

So, we described these results as following sentences. (**page 11, line 1**)

“The result of the predicting the GON has shown the 79.4% accuracy from multivariate logistic regression model.”
